# Emerging microbiome–mitochondria crosstalk in host defense and infectious diseases: mechanistic insights into NLRP3 inflammasome activation and mtDNA-mediated immunomodulation

**DOI:** 10.3389/fcimb.2026.1866924

**Published:** 2026-08-10

**Authors:** Xinxing Lu, Wenbin Sun, Daowei Zhang, Bin Hou, Huiyu Tai, Wenqing Yu, Xuehua Pu

**Affiliations:** 1 Department of Critical Care Medicine, The Affiliated Taizhou People’s Hospital of Nanjing Medical University, Taizhou, Jiangsu, China; 2 Department of Infectious Diseases, The Affiliated Taizhou People’s Hospital of Nanjing Medical University, Taizhou, Jiangsu, China

**Keywords:** host defense, infectious diseases, microbiome–mitochondria, mtDNA-mediated immunomodulation, NLRP3 inflammasome

## Abstract

Recent studies highlight a complex interaction between the gut microbiome and host mitochondrial dynamics in the modulation of immune responses as well as susceptibility to infectious and inflammatory pathologies. Recent empirical studies elucidate that metabolites derived from microbiota, encompassing short-chain fatty acids, trimethylamine, and indole derivatives, orchestrate mitochondrial functionality and the production of reactive oxygen species, which subsequently affect NLRP3 inflammasome activation. Dysbiosis within the gut microbiota has been documented to aggravate mitochondrial stress in host intestinal epithelial cells and other tissue-resident cells, facilitate the release of mitochondrial DNA (mtDNA), and initiate inflammatory pathways across a spectrum of conditions, including colitis, neurodegeneration, cardiovascular disorders, and sepsis. In contrast, the application of probiotics, postbiotics, and phytochemicals may restore microbial equilibrium, bolster mitochondrial integrity through mitophagy and PINK1/Parkin pathways, and mitigate NLRP3 inflammasome-mediated pyroptosis. Furthermore, experimental findings suggest that mtDNA functions as a damage-associated molecular pattern, activating cGAS-STING and NLRP3 signaling pathways, thereby establishing a connection between alterations in microbiota and systemic inflammation. The microbiome–mitochondria axis has been further associated with organ-specific immune responses, encompassing interactions among the gut-lung, gut-brain, gut-kidney, and gut-liver systems. Notably, the liver serves as a primary intermediary hub, receiving gut-derived metabolites and inflammatory mediators through the portal circulation and thereby linking intestinal microbial signals to peripheral immune and metabolic responses. Collectively, these investigations emphasize the emerging mechanistic significance of microbiota-induced modulation of mitochondrial function in host defense mechanisms, thereby illuminating potential therapeutic approaches that focus on microbial composition, mitochondrial dynamics, and inflammasome signaling to alleviate infectious and inflammatory pathologies. This review integrates contemporary understandings of the interactions between microbiota, mitochondria, and NLRP3 inflammasome activation, thereby establishing a framework for prospective translational research.

## 1 Introduction

The mammalian host engages in an ongoing interaction with its indigenous microbiota, a multifaceted ecological consortium that significantly influences immune equilibrium, metabolic processes, and susceptibility to diseases (Ruff et al., 2020). In recent years, this interaction has evolved beyond traditional host–microbe communication to encompass a dynamic interplay between the microbiome and mitochondria—organelles of prokaryotic lineage that preserve essential immunological and metabolic characteristics (Lee et al., 2024). This microbiome–mitochondria axis has emerged as a pivotal regulator of host defense mechanisms, incorporating microbial-derived signals with intracellular metabolic and danger-associated pathways (Benameur et al., 2025). Microbial metabolites such as short-chain fatty acids (SCFAs), secondary bile acids, and tryptophan derivatives modulate mitochondrial functionality by influencing oxidative phosphorylation, reactive oxygen species (ROS) production, and mitochondrial dynamics, thereby affecting the activation of immune cells and the ensuing inflammatory responses (Levy et al., 2017; Mills et al., 2017). In contrast, signals indicative of mitochondrial stress including mitochondrial ROS (mtROS), exposure of cardiolipin, and the release of mitochondrial DNA (mtDNA), function as essential mediators that connect cellular damage to the activation of the innate immune system (West et al., 2011; Riley and Tait, 2020). Within this conceptual framework, the NLRP3 inflammasome has garnered significant scholarly interest as a fundamental molecular entity that connects mitochondrial dysfunction with microbiome-derived signals. NLRP3 functions as a cytosolic receptor, exhibiting responsiveness to an extensive array of pathogen-associated molecular patterns and damage-associated molecular patterns (DAMPs), which culminates in the activation of caspase-1, the maturation of interleukin-1β (IL-1β) and IL-18, and the induction of pyroptotic cell death (Guo et al., 2015). Mitochondria assume a central role in the activation of NLRP3, as mitochondrial dysfunction facilitates the accumulation of mitochondrial reactive oxygen species (mtROS) and the cytosolic liberation of oxidized mtDNA, both of which serve as potent agonists for NLRP3 (Shimada et al., 2012; Mishra et al., 2021). Notably, the microbiome exerts influence over these mechanisms at various levels: commensal bacteria can mitigate excessive activation of the inflammasome through metabolic modulation, whereas dysbiosis exacerbates mitochondrial stress and predisposes the host to heightened inflammatory responses during infectious challenges (Belkaid and Hand, 2014; Zheng et al., 2020).

Despite significant progress in the field, the existing body of literature continues to exhibit fragmentation. Current reviews predominantly focus on either microbiome–immune interactions or the mitochondrial regulation of inflammation in a segregated manner, with minimal integration of these distinct domains. For example, reviews centered on gut microbiota primarily emphasize microbial metabolites, epithelial barrier integrity, and immune regulation while providing limited discussion of mitochondrial signaling pathways. Conversely, reviews addressing mitochondrial dysfunction and inflammasome activation frequently focus on mtROS generation, mitophagy, and mitochondrial quality control without adequately considering the regulatory influence of microbial ecosystems and microbiota-derived metabolites. Furthermore, although numerous investigations have elucidated the role of NLRP3 in the context of infectious diseases, they frequently neglect the mechanistic involvement of mtDNA as an immunomodulatory signal influenced by microbial ecosystems. Importantly, previous reviews seldom consolidate the manner in which microbiome-derived metabolites affect mitochondrial integrity to modulate mtDNA release and the subsequent activation of inflammasomes across varying infectious scenarios. This knowledge gap is particularly important because mitochondrial signals, including mtDNA, participate in multiple immunoregulatory pathways beyond inflammasome activation, including interferon responses and trained immunity (Nakayama and Otsu, 2018; Zhong et al., 2018). Another constraint within this domain pertains to the inadequate examination of bidirectional feedback mechanisms. Although it is firmly established that mitochondria play a regulatory role in immune responses, a limited number of investigations rigorously analyze the ways in which inflammasome activation and inflammation induced by infection mutually influence mitochondrial functionality and modify the microbiome. This neglect has impeded a comprehensive understanding of host defense systems, particularly in multifaceted diseases where metabolic, microbial, and immune pathways intersect. Moreover, variability in methodologies from *in vitro* systems devoid of microbial intricacy to *in vivo* models with restricted translational applicability, has exacerbated discrepancies in the elucidated mechanisms, highlighting the necessity for a more integrative and critical synthesis of the existing evidence.

The current review is consequently essential to reconcile these conceptual and methodological discrepancies by establishing a cohesive framework that interrelates microbiome composition, mitochondrial dynamics, and the activation of the NLRP3 inflammasome through the perspective of mtDNA-mediated immunomodulation. In contrast to prior reviews, this manuscript underscores mechanistic convergence, elucidating how microbial signals modulate mitochondrial integrity and how molecules derived from mitochondria subsequently influence immune responses during infections. By synthesizing perspectives from immunology, microbiology, and mitochondrial biology, this review aspires to transcend mere descriptive correlations to achieve a more causal and translational comprehension of host defense mechanisms. From a pragmatic standpoint, the accumulation of empirical data derived from both experimental and clinical investigations indicates that the modulation of the microbiome–mitochondria axis may provide innovative therapeutic avenues. Interventions such as the alteration of microbiota composition, administration of mitochondrial antioxidants, and application of inflammasome inhibitors have demonstrated potential in preclinical models; however, their translation into clinical practice is hindered by a limited comprehension of the intricate mechanisms that interlink these factors. A more sophisticated understanding of mtDNA signaling and its modulation by microbial and metabolic determinants could facilitate the formulation of precision medicine approaches tailored to the unique inflammatory and infectious profiles of individual patients.

Beyond their role as mere by-products of oxidative metabolism, mtROS function as pivotal signaling entities that modulate inflammatory pathways and innate immune activation. Excessive mtROS production can induce mitochondrial dysfunction by damaging mitochondrial proteins, lipids, and nucleic acids, thereby compromising mitochondrial integrity. Furthermore, mtROS-mediated mitochondrial injury promotes the release of mtDNA into the cytosol, where it acts as a DAMP capable of activating both the cGAS–STING pathway and the NLRP3 inflammasome. Through these interconnected mechanisms, mtROS and mtDNA cooperatively amplify inflammatory signaling and downstream immune responses. Emerging evidence further suggests that this mtROS–mtDNA signaling network contributes to microbiota-driven inflammation and immune dysregulation across multiple gut–organ axes, including the gut–liver, gut–lung, and gut–brain systems (Park et al., 2025; Adu et al., 2026). In conclusion, the interplay between the microbiome and mitochondria constitutes a pivotal yet inadequately investigated dimension of host defense mechanisms. Fundamental insights derived from existing literature and empirical observations encompass: (i) mitochondrial signaling pathways, notably mtDNA and mtROS, serve as crucial integrators of both microbial and host-derived stimuli within the framework of innate immunity; (ii) the NLRP3 inflammasome acts as a critical convergence point that associates dysbiosis with inflammatory pathology; (iii) the reciprocal interactions between the microbiota and mitochondria are indispensable for the preservation of immune homeostasis, yet these interactions tend to become maladaptive in the context of infection; and (iv) the therapeutic modulation of this intricate axis necessitates a comprehensive, systems-level perspective rather than the isolated adjustment of individual components. Addressing these challenges will be paramount for the successful translation of mechanistic insights into efficacious interventions for infectious and inflammatory diseases.

## 2 The gut microbiome and host immunity

### 2.1 Composition and functional roles of gut microbiota

The gut microbiota is a highly dynamic microbial ecosystem composed of bacteria, viruses, fungi, and archaea whose composition and functional capacity are shaped by diet, host genetics, environmental exposures, and medication use. Recent evidence suggests that microbial function and metabolite production may be more informative than taxonomic composition alone in predicting immune and metabolic outcomes (Mollica et al., 2017; Rose et al., 2018; Paparo et al., 2021; Cavaliere et al., 2022; Nicese et al., 2023; Zachos et al., 2024). Rather than existing as a static entity, the microbiota operates as a metabolically active organ that perpetually engages with host tissues, particularly interfacing with the immune system. This interaction commences early in the lifespan and is imperative for the appropriate development and maturation of both innate and adaptive immunity (Belkaid and Hand, 2014). Functionally, the gut microbiota plays a pivotal role in the maintenance of immune homeostasis through a variety of mechanisms. It modulates the differentiation of immune cell populations, including regulatory T cells (Tregs) and T helper 17 (Th17) cells, which are essential for sustaining the equilibrium between immune tolerance and inflammatory responses. Specific commensal bacteria, including *Bacteroides fragilis* and various Clostridium species, are known to facilitate the expansion of Tregs and the production of anti-inflammatory cytokines, while segmented filamentous bacteria are instrumental in eliciting Th17 responses, which are crucial for mucosal defense yet may contribute to pathological conditions when dysregulated (Ivanov et al., 2009; Round and Mazmanian, 2009). Beyond their influence on immune cell function, the microbiota is pivotal in sustaining the integrity of the intestinal barrier. It engenders the synthesis of mucus, antimicrobial peptides, and tight junction proteins, thus constraining pathogen ingress and the systemic circulation of microbial products (Hooper et al., 2012). Perturbation of this barrier, frequently linked with dysbiosis, results in heightened permeability and the translocation of microbial constituents such as lipopolysaccharide (LPS), which can incite systemic inflammation and activate innate immune pathways, including the NLRP3 inflammasome. This underscores the necessity for integrative methodologies that amalgamate compositional and functional analyses to enhance the understanding of microbiota-mediated immune regulation.

### 2.2 Microbiota-derived metabolites in immune modulation

**Figure 1:**
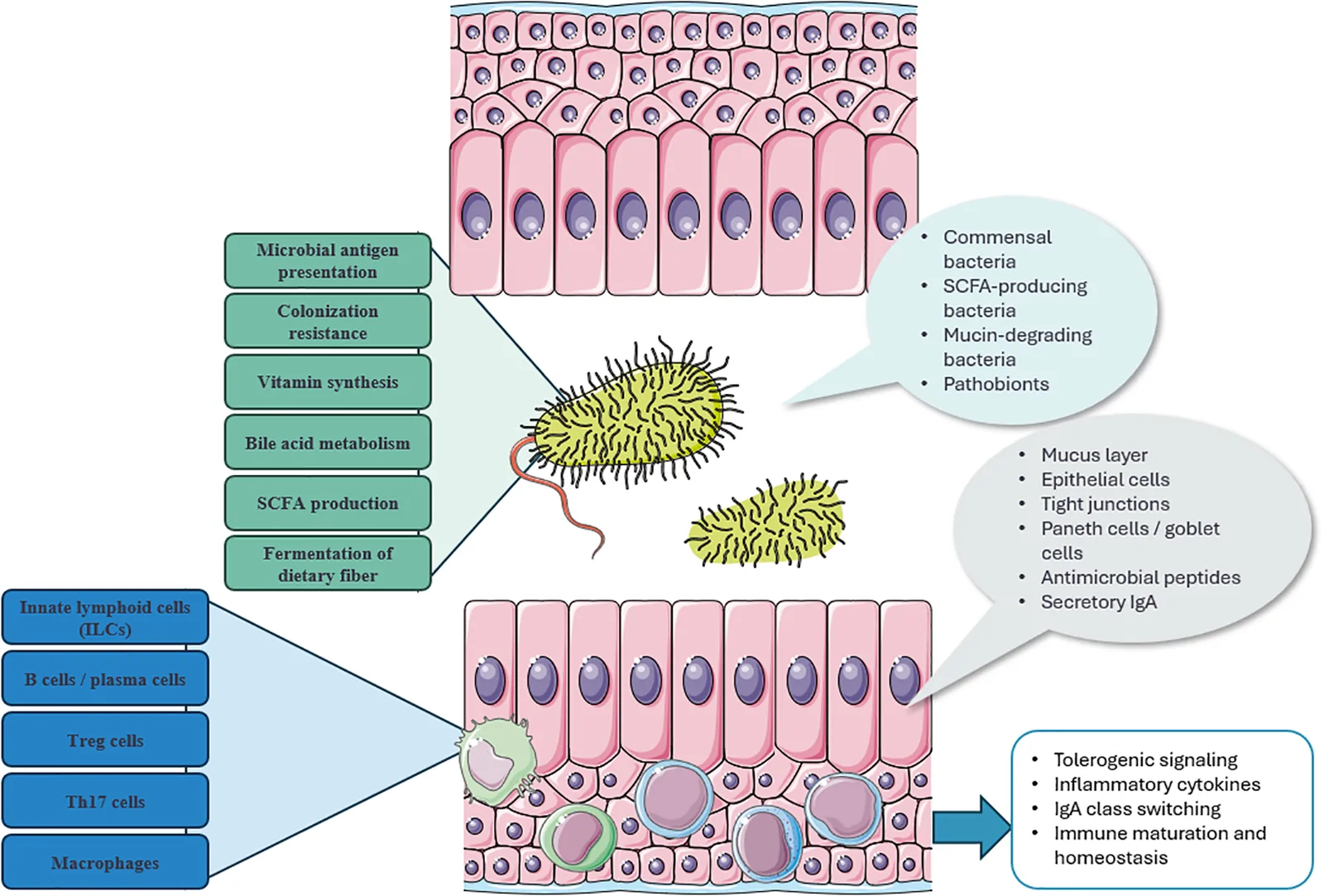
Interactions between the gut microbiome, intestinal barrier, and host immunity. The gut lumen contains diverse microbial communities, including commensal bacteria, SCFA-producing taxa, mucin-degrading microbes, and pathobionts. These microbes carry out key metabolic and ecological functions—such as fermentation of dietary fiber, SCFA production, bile acid metabolism, vitamin synthesis, colonization resistance, and microbial antigen presentation—that shape the mucosal environment. Signals derived from microbial activity influence epithelial barrier components, including the mucus layer, epithelial cells, tight junctions, Paneth and goblet cells, secretion of antimicrobial peptides, and production of secretory IgA. Beneath the epithelium, microbial products and metabolites modulate mucosal immune populations, including innate lymphoid cells (ILCs), B cells and plasma cells, Treg cells, Th17 cells, and macrophages. These interactions collectively regulate tolerogenic signaling, inflammatory cytokine production, IgA class switching, and overall immune maturation and homeostasis.

Microbiota-derived metabolites constitute significant mediators through which the gut microbiome exerts systemic influences on host immunity. Among these metabolites, SCFAs are synthesized through the fermentation of dietary fibers and have been extensively investigated for their immunomodulatory characteristics. SCFAs affect immune responses by activating G protein-coupled receptors (including GPR41 and GPR43), inhibiting histone deacetylases, and modulating cellular metabolism. These mechanisms collectively facilitate the differentiation of Tregs, diminish pro-inflammatory cytokine production, and improve epithelial barrier integrity (Furusawa et al., 2013; Koh et al., 2016). Among SCFAs, butyrate is the most thoroughly delineated metabolite concerning host immunometabolism and mitochondrial modulation (Cavaliere et al., 2022; Nicese et al., 2023; Zachos et al., 2024). Butyrate functions as a principal energetic substrate for intestinal epithelial cells and facilitates mitochondrial β-oxidation and oxidative phosphorylation, thereby endorsing epithelial homeostasis and barrier stability (Mollica et al., 2017; Cavaliere et al., 2022). Mechanistically, butyrate augments mitochondrial respiration by enhancing electron transport chain functionality while mitigating electron leakage and excessive ROS production (Cavaliere et al., 2022; Nicese et al., 2023). Empirical investigations have substantiated that butyrate enhances the operational efficiency of complexes I and III within the electron transport chain, culminating in increased ATP synthesis and diminished oxidative stress. Moreover, butyrate fortifies tight junction integrity, constrains intestinal permeability, and fosters immune tolerance via the proliferation of regulatory T cells and the inhibition of pro-inflammatory cytokine synthesis (Paparo et al., 2021; Nicese et al., 2023; Zachos et al., 2024). Through these interrelated impacts on mitochondrial metabolism, redox equilibrium, and immune regulation, butyrate emerges as a pivotal mediator that links the gut microbiota to host immune homeostasis and inflammatory modulation (Mollica et al., 2017; Rose et al., 2018; Paparo et al., 2021; Cavaliere et al., 2022; Nicese et al., 2023; Zachos et al., 2024). In addition to SCFAs, secondary bile acids produced via microbial metabolism of primary bile acids are instrumental in modulating immune responses. These metabolites engage with host receptors such as FXR and TGR5, thereby affecting macrophage polarization, dendritic cell activation, and cytokine secretion profiles (Hang et al., 2019). In a similar vein, metabolites derived from tryptophan, encompassing indole derivatives, engage the aryl hydrocarbon receptor (AHR), which plays a pivotal role in the regulation of mucosal immunity, epithelial integrity, and antimicrobial defense mechanisms (Zelante et al., 2013). Of significant importance, these metabolites further influence intracellular signaling cascades associated with mitochondrial functionality and inflammatory processes. SCFAs, particularly butyrate, enhance mitochondrial bioenergetics by promoting oxidative phosphorylation, improving electron transport chain efficiency, and limiting mtROS generation, consequently curtailing the release of pro-inflammatory mediators such as mtDNA. Reduced mtROS production decreases mitochondrial damage and may limit downstream activation of NLRP3 inflammasome signaling. Conversely, metabolic alterations associated with dysbiosis may instigate mitochondrial dysfunction, which in turn fosters heightened oxidative stress and the activation of inflammasome pathways, particularly the NLRP3 inflammasome (Levy et al., 2017; Mills et al., 2017). Moreover, the heterogeneity present in experimental systems and dietary contexts exacerbates the complexity of interpreting the resulting findings. A significant lacuna persists in establishing a direct correlation between distinct metabolite profiles and specific immunological outcomes in human pathology. To effectively address these constraints, it will be imperative to employ integrative multi-omics methodologies alongside clinically pertinent models to thoroughly elucidate the mechanisms by which microbiota-derived metabolites govern immune functionality (**Figure 1**).

## 3 Mitochondrial function and immunoregulation

Mitochondria are central regulators of immune function, integrating metabolic, redox, and danger-associated signals to coordinate both innate and adaptive immune responses. Mitochondrial activities encompass multiple interconnected processes, including ROS production, mitochondrial quality control through mitophagy, mtDNA-mediated signaling, and dynamic remodeling through fission and fusion events. Immune cells exhibit a dynamic alteration of mitochondrial architecture in reaction to pathogenic threats and inflammatory signals, with these modifications in dynamics significantly affecting cytokine synthesis, antigen presentation, and cellular survival pathways (Zhong et al., 2018). Disruptions in mitochondrial dynamics can influence innate sensor signaling pathways, such as the assembly of the NLRP3 inflammasome, thereby impacting host defense mechanisms and vulnerability to infections (Weinberg et al., 2015). Importantly, mitochondria function as central hubs for the integration of metabolic and immunological signals, orchestrating responses via bioenergetic adjustments and redox equilibrium (Pearce and Pearce, 2013). Abnormal mitochondrial dynamics have been associated with hyperinflammatory conditions and compromised pathogen eradication in models of bacterial, viral, and parasitic infections (Mills and O'Neill, 2016). Considering their critical function in immune modulation, the therapeutic alteration of mitochondrial dynamics has emerged as a potentially effective approach to reestablish immune responses in the context of infectious and inflammatory pathologies (Turrens, 2003).

### 3.1 Mitochondrial ROS production and signaling

mtROS function as pivotal signaling mediators that influence the functionality of immune cells and the activation of inflammasomes. The production of mtROS predominantly occurs at the electron transport chain complexes I and III during the process of cellular respiration (Sena and Chandel, 2012) and is augmented in response to signals associated with pathogens (Bulua et al., 2011). Within the realm of innate immunity, mtROS amplify the NF-κB and MAPK signaling pathways, thereby enhancing the production of pro-inflammatory cytokines and bolstering antimicrobial defenses (Zhou et al., 2011). Notably, mtROS serve as primary initiators of NLRP3 inflammasome formation, facilitating the activation of caspase-1 and the maturation of IL-1β/IL-18 (Dinarello, 2018). Nevertheless, an overabundance of mtROS can intensify tissue injury and perpetuate chronic inflammation, thereby implicating redox imbalance in conditions such as sepsis, viral infections, and autoimmune disorders (Schieber and Chandel, 2014). Consequently, the regulation of antioxidants is of paramount importance to ensure that mtROS contribute to the clearance of pathogens while minimizing harm to the host (Pickrell and Youle, 2015).

### 3.2 Mitophagy and PINK1/parkin pathways

Mitophagy—an autophagic process that selectively eliminates damaged mitochondria—is indispensable for preserving mitochondrial integrity and averting inappropriate immune activation. The PINK1/parkin signaling cascade coordinates the identification and elimination of depolarized mitochondria: PINK1 accumulates on compromised outer membranes and recruits parkin to facilitate ubiquitination and the formation of autophagosomes (Zhong et al., 2016). Effective mitophagy mitigates the accumulation of damaged mitochondria that could otherwise liberate pro-inflammatory mediators, including mtROS and mtDNA (Saitoh et al., 2008). Deficiencies in PINK1/parkin-mediated mitophagy have been correlated with increased activation of the NLRP3 inflammasome, elevated release of IL-1β, and amplified inflammatory responses in experimental infection models (Harris et al., 2011). For example, studies in experimental sepsis have demonstrated that impaired PINK1/Parkin-dependent mitophagy promotes the accumulation of dysfunctional mitochondria, resulting in excessive mtROS generation, enhanced inflammatory responses, and aggravated tissue injury. Conversely, restoration of PINK1/Parkin-mediated mitophagy alleviates mitochondrial damage and inflammatory lung injury in septic models (Chen et al., 2021). Similarly, evidence from viral infection models indicates that defective mitophagy contributes to sustained innate immune activation and tissue damage through the persistence of damaged mitochondria and dysregulated inflammatory signaling (Fu et al., 2022). In macrophages and dendritic cells, functional mitophagy maintains immune homeostasis, whereas compromised clearance instigates inflammasome-dependent pyroptosis (Shimada et al., 2012). Consequently, mitophagy plays a pivotal role in regulating the equilibrium between host defense mechanisms and inflammatory pathology.

### 3.3 Mitochondrial stress and mtDNA release

**Figure 2:**
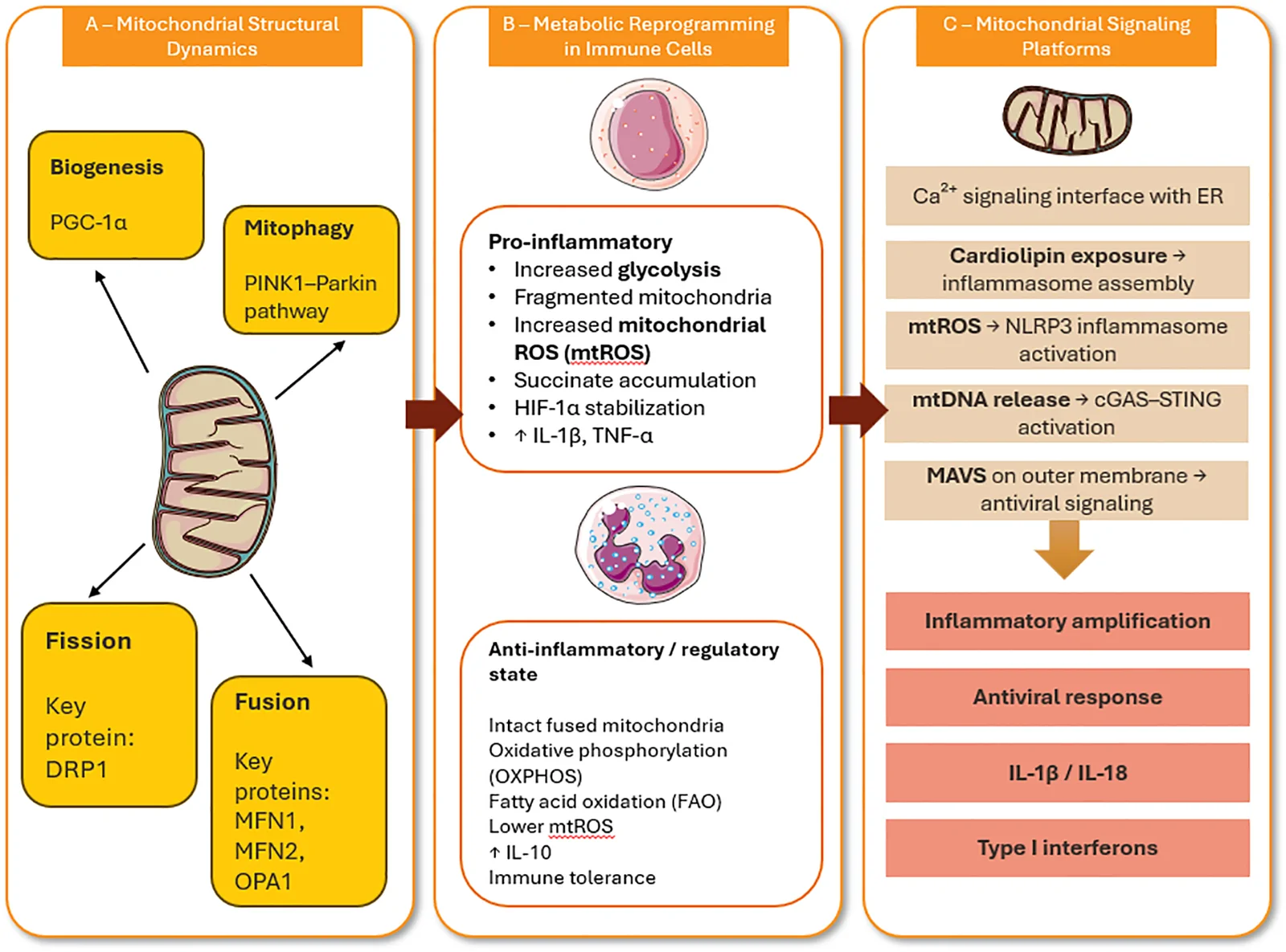
Mitochondrial dynamics orchestrate immune cell metabolism and signaling. Mitochondrial fusion, fission, mitophagy, and biogenesis regulate metabolic reprogramming in immune cells. Fragmented mitochondria are associated with glycolytic, pro-inflammatory phenotypes, increased mtROS production, and inflammasome activation. In contrast, fused mitochondrial networks support oxidative phosphorylation, fatty acid oxidation, and regulatory immune responses. Additionally, mitochondria serve as signaling platforms for MAVS-mediated antiviral responses and mtDNA-driven cGAS–STING and inflammasome activation, ultimately shaping immune homeostasis or inflammation.

Mitochondrial stress, which may arise from factors such as infection, oxidative damage, or metabolic dysregulation, can precipitate the permeabilization of mitochondrial membranes, consequently resulting in the release of mtDNA into the cytosolic and extracellular environments (West et al., 2015). Cytosolic mtDNA operates as DAMPs that activates sensors, including cGAS-STING and TLR9, thereby eliciting type I interferon responses and inflammatory signaling pathways (Nakahira et al., 2011). Concurrently, the accumulation of mtDNA fosters the activation of the NLRP3 inflammasome through either direct or indirect mechanisms, culminating in the activation of caspase-1 and the maturation of pro-inflammatory cytokines (Zhang et al., 2010). The presence of extracellular mtDNA additionally serves to amplify inflammatory responses by interacting with neighboring immune cells (Rongvaux et al., 2014). Given that mtDNA exhibits structural similarities to bacterial DNA, including the presence of hypomethylated CpG motifs, it can be recognized by pattern-recognition receptors such as TLR9. This molecular mimicry enables released mtDNA to trigger innate immune pathways that normally respond to microbial nucleic acids, thereby promoting inflammatory cytokine production and amplifying host immune responses. Consequently, mtDNA serves as a critical link between mitochondrial damage and pathogen-sensing mechanisms, shaping the progression of infectious and inflammatory diseases (**Figure 2**).

## 4 NLRP3 inflammasome activation in microbiome–mitochondria axis

### 4.1 Evidence from preclinical and clinical studies

Accumulating evidence from preclinical and translational studies supports a central role for microbiota–mitochondria–inflammasome interactions in the pathogenesis of inflammatory, metabolic, neurological, cardiovascular, and infectious diseases. Across multiple DSS-induced colitis models, interventions including traditional medicinal formulations and probiotic administration consistently attenuated disease severity by restoring microbial homeostasis, enhancing epithelial barrier integrity, suppressing oxidative stress, and limiting NLRP3 inflammasome activation (Wang et al., 2025; Mao et al., 2026). Despite differences in therapeutic agents, these studies converged on a common mechanistic framework involving modulation of the NOX2/ROS/mtDNA/NLRP3 signaling axis and improvement of mitochondrial homeostasis (Wang et al., 2025; Mao et al., 2026) (**Table 1**). Similar observations have been reported in gut–lung axis models. In LPS-induced acute lung injury, restoration of gut microbial balance increased SCFA production, improved intestinal barrier function, reduced mitochondrial dysfunction, and suppressed activation of the TLR4/NF-κB/NLRP3 pathway, ultimately attenuating systemic inflammation and tissue injury (Zhu et al., 2026).

**Table 1:** Summary of gut microbiota- and metabolite-mediated regulation of NLRP3 inflammasome across multiple disease models.

Model/subjects	Intervention	Mechanism involving NLRP3	Other molecular/pathway highlights	Gut microbiota/metabolomics findings	Ref
DSS-induced colitis mice; LPS-induced HT-29 cells	Xu Chunfu’s Modified Xianglian Pill (XXLP)	Suppression of NLRP3 inflammasome via NOX2/ROS/mitochondrial pathway	↓ IL-1β, IL-18, TNF-α, IL-6; proteomics identified NOX2 as key target; validated by WB, qRT-PCR, IF, TEM	↑ Muribaculaceae & Ruminococcaceae; ↓ Enterobacteriaceae; gut microbiota correlated with NOX2-related protein expression	(Mao et al., 2026)
LPS-induced acute lung injury (ALI) rats; cell experiments	Houttuynia cordata Thunb. essential oil (HEO)	Inhibition of NLRP3 inflammasome via decreased mitochondria-ER contacts and mitofusin 2	↓ IL-1β, suppression of TLR4/NF-κB p65 signaling; strengthened intestinal barrier (↑ ZO-1)	Restored gut microbiota dysbiosis; ↑ SCFAs; tryptophan metabolism linked to Bacteroides & Lactobacillus	(Zhu et al., 2026)
D-gal/AlCl3-induced Alzheimer’s disease rats	Postbiotics from B. animalis IOBL07, L. plantarum IOB602, L. paracasei IOB413	Suppressed NLRP3 inflammasome via TLR4/MyD88/NLRP3 signaling	Reduced microglial activation, Aβ accumulation; ameliorated oxidative stress; improved mitochondrial function	↑ Lachnospiraceae, Ruminococcus, Lactobacillus; ↓ Muribaculaceae; ↑ fecal SCFAs; 11 metabolites enriched in IOB602 group (e.g., indole derivatives)	(Jia et al., 2025)
DSS-induced colitis piglets	Probiotic B. velezensis MZ09	Suppresses NLRP3-mediated pyroptosis via STAT3/HIF-1α axis	↑ IL-10; improved tight junction proteins (Claudin-1, Occludin, ZO-1); mitigated mitochondrial damage	↑ Firmicutes & Lactobacillus; ↓ Proteobacteria & Escherichia-Shigella; ↑ SCFAs activating GPR43-STAT3	(Wang et al., 2025)
ApoE-/- mice with HFD & choline-induced AS	Buyang Huanwu Decoction	Inhibition of NLRP3 via TLR4/NF-κB pathway	↓ ROS, ↓ inflammatory cytokines, modulated mitophagy (LC3, NIX, BNIP3, PINK1/Parkin); ↑ GPR41, GPR43, FXR, TGR5	Regulated gut microbiota composition; ↓ TMAO levels; LC-MS/MS-detected active components	(Chang et al., 2025)
Mice & human macrophage cell model	AFB1 exposure; rescued with AHR inhibitor or antioxidants	↑ NLRP3 via AHR-TLR4/p-STAT3 (Ser727) mitochondrial oxidative stress	↑ ICAM-1 & IDO; macrophage M1 polarization; ROS-dependent	Gut barrier & microbiota severely damaged	(Zhang et al., 2024)
GVHD mice; BMDMs	TMAO or high-choline diet; 3,3-dimethyl-1-butanol reversed	↑ NLRP3 required for M1 macrophage polarization	↑ IL-1β, IL-6, TNF-α, CXCL9, CXCL10; ↑ mitochondrial ROS & NF	Not directly microbiota, but TMAO is gut microbial metabolite	(Wu et al., 2020)
Rats under chronic unpredictable mild stress (CUMS)	Fecal microbiota transplantation (FMT) & probiotics	Suppression of NLRP3 inflammasome in brain and colon	↓ Caspase-1, ASC; restored tight junction proteins (Occludin, ZO-1)	FMT altered gut microbiota; probiotics improved microbial composition	(Huang et al., 2023)
Aged rats; FMT to young hosts; clinical cohort	FMT & NLRP3 inhibition (MCC950)	↑ NLRP3 via LPS & glucose; inflammasome activation promotes atrial fibrosis	↑ circulating LPS & glucose; atrial fibrosis dependent on NLRP3	Gut dysbiosis transmitted via microbiota-intestinal barrier-atria axis	(Zhang et al., 2022)
UOX-knockout HUA rats; FMT in mice	HUA-induced gut dysbiosis	Activation of NLRP3 inflammasome	Renal injury, fibrosis, impaired intestinal barrier	Altered gut microbiota; accumulation of gut-derived uremic toxins	(Zhou et al., 2024)
H9c2 rat cardiomyocytes; C57BL/6 J mice; NLRP3/MLKL/RIPK3 KO mice	Doxorubicin (DOX)	NLRP3 mediates cardiotoxicity via inflammasome activation	↑ LC3I/II; cardiomyocyte apoptosis; inflammatory cytokines	DOX altered gut microbiota; NLRP3 KO modulated abundance of Akkermansia muciniphila	(Yin et al., 2025)
Intestinal ischemia/reperfusion (II/R) mice; BMDMs	B. longum & indole-3-carboxaldehyde (3-IAld)	Inhibits macrophage NLRP3 inflammasome via HDAC3	HDAC3 translocation to mitochondria; regulates mitochondrial FAO	B. longum restored gut microbiota diversity; increased 3-IAld levels	(Miao et al., 2025)
Elderly septic mice (CLP model); FMT & acetic acid intervention	Young-donor FMT & acetic acid supplementation	Inhibition of NLRP3 inflammasome via FFAR2 signaling	↑ plasma and gut acetic acid; FFAR2 knockdown abrogates effects	FMT restored microbiota diversity; ↑ Akkermansia; ↑ fecal acetic acid	(Yu et al., 2025)

DAI, Disease Activity Index; DSS, Dextran Sulfate Sodium; ALI, Acute Lung Injury; FMT, Fecal Microbiota Transplantation; SCFAs, Short-Chain Fatty Acids; TMAO, Trimethylamine N-oxide; HUA, Hyperuricaemia; II/R, Intestinal Ischemia/Reperfusion; AD, Alzheimer’s Disease; DOX, Doxorubicin; BMDM, Bone Marrow-Derived Macrophages; FFAR2, Free Fatty Acid Receptor 2; ROS, Reactive Oxygen Species; ER, Endoplasmic Reticulum; HFD, High-Fat Diet; ApoE-/-, Apolipoprotein E knockout; NOX2, NADPH Oxidase 2; HEO, Houttuynia cordata Essential Oil; HDAC3, Histone Deacetylase 3; BYHWD, Buyang Huanwu Decoction; MZ09, Bacillus velezensis MZ09; ASC, Apoptosis-associated Speck-like Protein; NF-κB, Nuclear Factor-kappa B; STAT3, Signal Transducer and Activator of Transcription 3; HIF-1α, Hypoxia-Inducible Factor 1-alpha.

Evidence from neuroinflammatory and neurodegenerative disease models further highlights the importance of microbiota-derived metabolites in regulating mitochondrial function and inflammasome activity. Postbiotic interventions improved cognitive outcomes, reduced oxidative stress, and inhibited TLR4/MyD88/NLRP3 signaling while simultaneously increasing beneficial microbial taxa and SCFA production (Jia et al., 2025). Likewise, chronic stress-associated depressive phenotypes were transferable through fecal microbiota transplantation (FMT) and accompanied by enhanced NLRP3 activation and intestinal barrier disruption, whereas probiotic treatment reversed these alterations (Huang et al., 2023). Cardiovascular studies have similarly demonstrated an intimate connection between microbial metabolites, mitochondrial homeostasis, and inflammatory signaling. In atherosclerosis models, modulation of gut microbiota reduced circulating TMAO levels, suppressed TLR4/MyD88/NF-κB/NLRP3 signaling, and improved mitochondrial quality-control pathways (Chang et al., 2025). In age-related atrial fibrillation, dysbiotic microbiota increased circulating LPS and glucose levels, promoted NLRP3 activation, and accelerated atrial fibrosis, whereas microbiota restoration or pharmacological inhibition of NLRP3 mitigated disease progression (Zhang et al., 2022). Similar microbiota-dependent inflammatory mechanisms have also been implicated in doxorubicin-induced cardiotoxicity (Yin et al., 2025).

Additional studies in hepatic, renal, intestinal ischemia/reperfusion, graft-versus-host disease, and sepsis models have reinforced the concept that microbiota-derived metabolites act as key regulators of mitochondrial function and immune responses. TMAO promoted NLRP3-dependent macrophage polarization and inflammatory injury in graft-versus-host disease (Wu et al., 2020), whereas indole-derived metabolites and acetate exerted protective effects through modulation of mitochondrial metabolism, inflammasome activity, and epithelial barrier integrity (Miao et al., 2025; Yu et al., 2025). In hyperuricemia-associated renal injury and aflatoxin-induced steatohepatitis, dysbiosis exacerbated tissue damage through inflammatory pathways involving oxidative stress, mitochondrial dysfunction, and NLRP3 activation (Zhang et al., 2024; Zhou et al., 2024). Collectively, these studies reveal several recurring mechanistic themes across diverse disease models. First, dysbiosis-induced oxidative stress frequently converges on the NOX2/ROS/mtDNA/NLRP3 axis, promoting inflammasome activation and tissue injury. Second, disruption of intestinal barrier integrity facilitates microbial translocation and activation of TLR4/NF-κB-dependent inflammatory pathways. Third, microbiota-derived metabolites, including SCFAs, indole derivatives, acetate, and TMAO, regulate mitochondrial homeostasis and immune responses through interconnected metabolic and inflammatory signaling networks.

### 4.2 Molecular and cellular mechanisms

#### 4.2.1 Metabolite regulation

Microbiota-derived metabolites represent key molecular mediators linking gut microbial composition to mitochondrial function and immune regulation. SCFAs, particularly butyrate and acetate, enhance mitochondrial oxidative metabolism, promote regulatory T-cell differentiation, and suppress excessive inflammatory responses through G-protein-coupled receptor signaling and epigenetic regulation (Wang et al., 2025; Yu et al., 2025). Similarly, indole derivatives generated from microbial tryptophan metabolism contribute to mucosal homeostasis and immune tolerance through AHR-dependent pathways while simultaneously influencing mitochondrial function and redox balance (Jia et al., 2025; Miao et al., 2025). In contrast, elevated levels of TMAO have been associated with increased mitochondrial oxidative stress, macrophage activation, and NLRP3 inflammasome signaling, thereby contributing to inflammatory pathology (Wu et al., 2020; Chang et al., 2025). Together, these observations indicate that microbial metabolites serve as critical intermediaries connecting microbial ecology with mitochondrial homeostasis and host immunity.

#### 4.2.2 Barrier function and microbial translocation

The intestinal barrier constitutes an essential interface between the microbiota and the host immune system. Dysbiosis frequently disrupts tight-junction proteins, including ZO-1, Occludin, and Claudin-1, resulting in increased intestinal permeability and systemic dissemination of microbial products such as lipopolysaccharide (Huang et al., 2023; Wang et al., 2025; Zhu et al., 2026). This process promotes activation of innate immune pathways, enhances systemic inflammation, and contributes to organ-specific pathology involving the intestine, lung, kidney, cardiovascular system, and central nervous system (Zhang et al., 2022; Huang et al., 2023; Zhou et al., 2024; Zhu et al., 2026). Conversely, probiotic administration, postbiotic supplementation, and FMT restore barrier integrity, reduce microbial translocation, and attenuate downstream inflammatory responses (Huang et al., 2023; Jia et al., 2025; Yu et al., 2025). These findings emphasize the central importance of barrier preservation in maintaining immune homeostasis and limiting inflammasome activation.

#### 4.2.3 Integration of mitochondrial and inflammasome signaling pathways

A consistent finding across disease models is the convergence of microbial signals on a limited number of conserved mitochondrial and inflammatory pathways. One major mechanism involves activation of the NOX2/ROS/mtDNA/NLRP3 axis, whereby dysbiosis-induced oxidative stress promotes mitochondrial damage, mtDNA release, and inflammasome activation (Zhang et al., 2024; Mao et al., 2026). A second recurring pathway involves TLR4/MyD88/NF-κB signaling, which links microbial products and barrier dysfunction to cytokine production and NLRP3 activation (Chang et al., 2025; Jia et al., 2025; Zhu et al., 2026). Additional regulatory pathways, including STAT3/HIF-1α signaling, FFAR2-mediated responses, HDAC3-dependent metabolic regulation, and PINK1/Parkin-mediated mitophagy, further integrate microbial-derived signals with mitochondrial quality control and immune modulation (Miao et al., 2025; Wang et al., 2025; Yu et al., 2025). Importantly, these pathways do not operate independently but rather form interconnected regulatory networks. Mitochondrial dysfunction enhances mtROS generation and mtDNA release, which amplify inflammatory signaling, whereas microbial metabolites can either exacerbate or attenuate these responses depending on their composition and abundance. This integrated signaling framework provides a mechanistic explanation for how microbiota-driven alterations influence organ-specific inflammation, tissue injury, and disease progression across diverse pathological settings.

## 5 mtDNA-mediated immunomodulation

### 5.1 Evidence from preclinical and clinical studies

An investigation assessed the ramifications of simultaneous exposure to LPS and zidovudine (ZDV) on the gut-brain axis and assessed the potential protective effects of probiotics. In murine models, the combination of LPS and ZDV induced systemic inflammation, cognitive deterioration, intestinal injury, and dysbiosis characterized by an increase in pro-inflammatory bacterial populations. Furthermore, this combination resulted in the elevation of neuroinflammatory biomarkers such as TNF-α within cerebral tissue. Administration of Bacillus subtilis effectively mitigated memory deficits, attenuated the expression of inflammatory genes (TNF-α, Il1b, Il6), and stimulated PINK1/PTEN-mediated mitophagy, all while maintaining mitochondrial integrity and microbial diversity. Conversely, a mixture of lactobacilli exacerbated intestinal damage, cognitive impairment, and inflammatory responses. Collectively, the findings indicate that LPS and ZDV disrupt the communication between the gut and brain; however, Bacillus subtilis demonstrates significant neuroprotective properties. These results underscore the critical role of probiotic strain specificity in addressing cognitive disorders associated with the gut-brain axis (Murgina et al., 2026) (**Table 2**). Another research explored the ramifications of trimethylamine (TMA), a metabolite originating from gut microbiota, on the functionality of intestinal epithelial cells utilizing Caco-2 cell lines. The exposure to TMA (1 mM) markedly elevated the levels of pro-inflammatory cytokines (IL-6, IL-1β), signifying an activation of the inflammatory response. Furthermore, TMA induced alterations in epigenetic regulation, resulting in the upregulation of SIRT1 and DNMT1, while concurrently downregulating DNMT3A. Notwithstanding the observed increase in SIRT1 gene expression, TMA diminished the overall activity of sirtuin enzymes in a dose-dependent manner. Evidence of mitochondrial dysfunction was apparent, characterized by a decrease in ATP production, a reduction in ND6 expression, and diminished mtDNA copy numbers, in conjunction with heightened methylation within the mtDNA D-loop region. Moreover, TMA was found to augment epithelial permeability, thereby indicating a compromise in barrier integrity. Importantly, although this study did not directly evaluate mtDNA release or cytosolic mtDNA accumulation, the observed reductions in mtDNA copy number, alterations in mtDNA methylation, impaired ATP production, and mitochondrial dysfunction suggest disruption of mtDNA homeostasis. These findings provide indirect evidence that TMA-induced mitochondrial injury may create conditions favorable for mtDNA-mediated inflammatory signaling, although direct mechanistic confirmation remains to be established (Bordoni et al., 2024).

**Table 2:** Summary of gut microbiota- and metabolite-driven mitochondrial DNA (mtDNA) modulation, epigenetic regulation, and cellular outcomes across experimental and clinical studies.

Model/system	Key findings	Mitochondrial/DNA involvement	Gut microbiota/metabolite role	Intervention/outcome	Ref
Mouse model of LPS + Zidovudine-induced neuroinflammation	LPS + ZDV caused systemic inflammation, cognitive deficits, intestinal damage, and neuroinflammation	cf-mtDNA release prevented by Bacillus subtilis; activation of PINK1/PTEN-dependent mitophagy	Dysbiosis with increased pro-inflammatory Muribaculaceae	Bacillus subtilis restored cognitive function, reduced Il1b/Il6/Tnfa, and normalized gut microbiota; Lactobacilli mixture ineffective	(Murgina et al., 2026)
*In vitro* human intestinal epithelial Caco-2 cells	TMA increased IL-6 and IL-1β, reduced ATP levels, decreased ND6 expression	Excess TMA reduced mtDNA copy number, increased D-loop methylation, disrupted mitochondrial homeostasis	Not directly studied but TMA is gut microbiota-derived metabolite	TMA excess promoted inflammation, epigenetic alterations, mitochondrial dysfunction, and barrier permeability	(Bordoni et al., 2024)
Dairy goats (SARA model) & mice recipients	High-concentrate diet caused rumen dysbiosis, LPS increase, systemic inflammation, and mastitis	Elevated mtDNA activated cGAS-STING → NF-κB/NLRP3 → TNF-α, IL-1β	Dysbiosis of rumen microbiota contributed to mtDNA-mediated inflammation	Rumen microbiota transplants from SARA goats induced mastitis; targeting gut microbiota suggested as therapeutic strategy	(Zhao et al., 2025)
Benthic fish (*Leiocassis longirostris*)	Florfenicol caused 43.65% reduction in microbial genes, 96.16% decrease in host mRNA, 69.11% increase in host DNA contamination, intestinal mucosal injury	Mitochondrial dysfunction, ROS metabolism, activation of chronic disease pathways (Parkinson’s, Huntington’s, diabetic cardiomyopathy)	Dysbiosis: loss of Cetobacterium, overgrowth of Clostridium and Acinetobacter; impaired metabolite synthesis (ascorbic acid, lovastatin acid)	Antibiotic exposure caused persistent microbiome dysfunction, structural intestinal damage, apoptosis, and increased ARG transfer risk	(Feng et al., 2025)
C57BL/6 mice + *in vitro* cells	High-choline diet increased TMAO, worsened arsenic-induced depressive behaviors, hippocampal damage, and astrocyte depletion	Mitochondrial dysfunction, oxidative stress, apoptosis, ferroptosis	TMAO (microbiota-derived metabolite) elevated by diet, enhanced arsenic accumulation in plasma and brain	PERK/eIF2α/ATF4 pathway activated; PERK knockdown attenuated apoptosis and ferroptosis	(Zhang et al., 2025)
Rats on high-fat diet	High-fat diet increased LPS and hepatic injury; butyrate alleviated liver damage	Improved mitochondrial function, reduced ROS	Butyrate reprogrammed intestinal DNA virome (phageome), augmented phage-bacteria interactions, reduced LPS-mediated damage	Fecal phage transplantation from butyrate-treated rats prevented liver injury, improved mitochondrial and ROS outcomes	(Zhang et al., 2025)
Mouse and cellular models	Age-associated increase in PAGln promotes host cellular senescence	Mitochondrial dysfunction and DNA damage via ADR-AMPK signaling	Gut microbiota metabolite PAGln elevated in older individuals	ADR blockade or senolytics therapy prevented PAGln-induced senescence	(Yang et al., 2025)
SH-SY5Y human neuroblastoma cells	HkRA decreased Aβ-induced cytotoxicity, apoptosis, and oxidative stress	Mitochondrial cytochrome c release reduced; DNA damage decreased	Inactivated probiotic cells (paraprobiotic) protected neurons	Upregulated antioxidative genes (HO-1, Nrf2, PKC-δ) and BDNF; decreased caspase-3 activity	(Choo et al., 2024)
Broiler chickens (embryo injection)	Prebiotics altered mitochondrial gene expression	mtDNA copy number stable; respiratory chain and energy metabolism genes up/down regulated	Prebiotics (XOS3, XOS4, MOS3, MOS4) modulated mitochondria via gut microbiota	XOS4 and MOS3 upregulated genes in cecal mucosa; all prebiotics downregulated genes in cecal tonsils; significant changes in CYCS, ND2, NRF1, TFAM	(Dunislawska et al., 2023)
SAMP8 mice	LcS improved muscle mass, strength, mitochondrial function, and reduced inflammation/ROS	Mitochondrial biogenesis and function restored; apoptosis, p53 signaling reduced; DNA repair and recombination enhanced	Lactobacillus casei Shirota modulated gut microbiota composition and SCFA production	High-dose LcS restored muscle function, mitochondrial activity, and favorable microbiota profile	(Chen et al., 2022)
Human subjects with metabolic syndrome	Allogenic FMT induced larger gut microbiota shifts than autologous; correlated with insulin sensitivity improvement	DNA methylation changes in peripheral blood mononuclear cells; AFAP1 gene methylation (mitochondrial function and insulin sensitivity)	Introduction of new gut microbes (e.g., Prevotella) modulated plasma metabolome and epigenome	Allogenic FMT improved metabolic and epigenetic profiles linked to mitochondrial function and insulin sensitivity	(van der Vossen et al., 2021)

LPS, Lipopolysaccharide; ZDV, Zidovudine; cf-mtDNA, Cell-Free Mitochondrial DNA; mtDNA, Mitochondrial DNA; TMA, Trimethylamine; TMAO, Trimethylamine N-oxide; SARA, Subacute Ruminal Acidosis; HCD, High-Concentrate Diet; RMT, Rumen Microbiota Transplantation; ROS, Reactive Oxygen Species; ER, Endoplasmic Reticulum; PERK, Protein Kinase RNA-like Endoplasmic Reticulum Kinase; SCFAs, Short-Chain Fatty Acids; ADR, Adrenoreceptor; AMPK, AMP-Activated Protein Kinase; BDNF, Brain-Derived Neurotrophic Factor; FMT, Fecal Microbiota Transplantation; LcS, Lactobacillus casei Shirota; SAMP8, Senescence-Accelerated Mouse Prone 8; AFAP1, Actin Filament Associated Protein 1.

The aim of a study was to examine the influence of the microbiota–gut–mammary axis in the etiology of mastitis utilizing a subacute ruminal acidosis-associated model provoked by a high-concentrate dietary regimen. Disruption of the microbiota and damage to the rumen epithelium resulted in elevated serum LPS concentrations, systemic inflammatory responses, and deterioration of the blood–milk barrier. Augmented levels of mtDNA were identified in both rumen and mammary tissues, thereby establishing a connection between gut dysbiosis and mammary inflammation. From a mechanistic perspective, mtDNA was found to activate the cGAS-STING signaling pathway, which consequently instigated NF-κB and NLRP3 activation, leading to an increase in pro-inflammatory cytokines, specifically TNF-α and IL-1β. The transplantation of rumen microbiota from affected subjects into mice elicited mastitis-like pathology, thereby confirming a causal relationship. Furthermore, mtDNA derived from inflamed mammary tissue elicited inflammatory responses in recipient organisms (Zhao et al., 2025). Another work evaluated the long-term implications of sub-therapeutic antibiotic administration within aquaculture, specifically targeting Chinese longsnout catfish subjected to florfenicol exposure. The intervention elicited pronounced intestinal dysbiosis, evidenced by a 43.65% reduction in microbial gene abundance and a 96.16% decline in host mRNA expression, coupled with heightened levels of host DNA contamination, signifying a collapse of the mucosal barrier. Beneficial microbial communities, exemplified by Cetobacterium, were supplanted by opportunistic genera such as Clostridium and Acinetobacter. Furthermore, functional metabolic pathways were inhibited, whereas genes associated with electron transport exhibited upregulation, correlating with mitochondrial dysfunction, oxidative stress, and pathways linked to chronic disease manifestations. The depletion of critical microbial metabolites exacerbated intestinal damage and apoptotic processes. Moreover, the risk of antibiotic resistance gene transfer escalated markedly (Feng et al., 2025).

A research examined the interplay between arsenic (As) exposure and TMAO concerning neurotoxicity and depressive disorders. Network toxicology analyses indicated that both and TMAO modulate pathways associated with apoptosis, ferroptosis, and ER stress. In murine models, a diet rich in choline resulted in elevated TMAO concentrations, which aggravated As-induced behaviors indicative of depression, alongside hippocampal impairment and loss of astrocytes. Empirical data corroborated that TMAO facilitated the accumulation of arsenic in both plasma and cerebral tissues. *In vitro* experiments demonstrated that TMAO heightened As-induced oxidative stress, DNA damage, mitochondrial dysfunction, apoptosis, and ferroptosis. Mechanistically, TMAO activated the PERK/eIF2α/ATF4 pathway associated with ER stress, whereas the silencing of PERK attenuated these detrimental effects. Although mtDNA release was not directly measured in this model, the observed mitochondrial dysfunction, oxidative stress, and DNA damage are recognized upstream events that may facilitate mtDNA instability and subsequent activation of mtDNA-sensitive inflammatory pathways. Therefore, these findings support a potential, albeit indirect, contribution of mtDNA-mediated immunomodulation to TMAO-associated neurotoxicity (Zhang et al., 2025). The aim of a work was to examine the significance of intestinal bacteriophages and their interactions with LPS in the context of high-fat diet-induced nonalcoholic fatty liver disease (NAFLD). An excessive intake of dietary fats resulted in elevated endogenous levels of LPS, which contributed to hepatic injury. The administration of butyrate, a metabolite produced by beneficial gut microbiota, mitigated hepatic damage by reconfiguring the intestinal virome, specifically by augmenting phage diversity. This reprogramming facilitated interactions between bacteriophages and bacterial LPS, resulting in enhanced mitochondrial functionality and diminished ROS, thereby conferring protection to hepatic cells. Fecal phage transplantation from butyrate-treated subjects further validated the protective role of phage–LPS interactions in attenuating liver injury (Zhang et al., 2025).

In a study, it was examined the role of gut microbiota in the aging process through its facilitation of cellular senescence. The researchers identified phenylacetylglutamine (PAGln), a metabolite derived from the microbiota, as a significant contributor to this phenomenon. Age-related modifications in gut microbiota resulted in an elevated synthesis of phenylacetic acid and its subsequent metabolite PAGln in elderly populations. Both *in vitro* and *in vivo* experimental models substantiated that PAGln promotes the expression of a senescent phenotype. Mechanistically, PAGln instigated mitochondrial dysfunction and DNA damage via the activation of the adrenoreceptor (ADR)–AMPK signaling pathway. Notably, the inhibition of ADRs or the implementation of senolytic therapies diminished PAGln-induced cellular senescence *in vivo.* While direct measurements of mtDNA release were not performed, PAGln-induced mitochondrial dysfunction and DNA damage suggest perturbation of mitochondrial genomic integrity. These alterations may contribute to mtDNA-dependent inflammatory signaling and represent a plausible mechanistic link between microbiota-derived metabolites and immunometabolic aging (Yang et al., 2025). Another investigation assessed the neuroprotective properties of heat-killed Ruminococcus albus (hkRA) in mitigating β-amyloid (Aβ)-induced cytotoxicity within SH-SY5Y neuronal cell lines, which serve as a paradigm for AD. Exposure to Aβ elicited oxidative stress, induced DNA damage, and precipitated apoptosis. Treatment with hkRA significantly ameliorated cytotoxicity and DNA impairment, while also modulating apoptotic signaling pathways by enhancing the bax/bcl-2 ratio and attenuating caspase-3 activation. Furthermore, it bolstered antioxidant defenses through the upregulation of HO-1, Nrf2, and PKC-δ, alongside augmenting the expression of brain-derived neurotrophic factor (BDNF). In addition, hkRA influenced mitochondrial functionality by preserving the distribution of cytochrome c. Collectively, these findings indicate that hkRA confers protection to neuronal cells against Aβ-induced oxidative stress and apoptosis, thereby underscoring its potential utility as a paraprobiotic in the prevention or management of neurodegenerative processes associated with AD (Choo et al., 2024).

In addition, a research evaluated the impact of prebiotics on mitochondrial functionality and gene expression in broiler chickens, thereby emphasizing the relationship between gut microbiota and mitochondrial systems. Prebiotics, specifically XOS and MOS, were administered via injection into eggs during the incubation phase, and subsequent analysis of mitochondrial parameters was conducted following hatching. The findings indicated that the mtDNA copy number exhibited stability within both cecal mucosa and tonsils. Nevertheless, the application of prebiotics resulted in a pronounced alteration of mitochondrial gene expression. In the cecal mucosa, XOS4 and MOS3 were observed to upregulate genes associated with energy metabolism and the functionality of the respiratory chain, including CYCS, ND2, NRF1, and TFAM. Conversely, all prebiotic treatments led to a downregulation of these genes in the cecal tonsils (Dunislawska et al., 2023). A research examined the impact of *Lactobacillus casei* Shirota (LcS) on age-associated sarcopenia utilizing senescence-accelerated murine models. LcS administration over a period of 12 weeks significantly enhanced muscle mass, strength, and mitochondrial functionality in elderly mice. Furthermore, it reinstated levels of SCFAs, diminished inflammation and ROS, and ameliorated overall metabolic health. The composition of the gut microbiota exhibited profound alterations, with an enrichment of beneficial bacterial genera correlated with improved muscular and physiological outcomes. Functional predictions indicated a decrease in apoptosis and p53 signaling, in conjunction with augmented DNA repair and protein synthesis pathways. These results imply that LcS alleviates age-related muscular degeneration by modulating the gut–muscle axis, enhancing mitochondrial function, lessening inflammation, and restructuring the gut microbiota, thereby underscoring its potential as a therapeutic intervention for sarcopenia (Chen et al., 2022). The purpose of a study was to determine the ramifications of FMT on the gut microbiome, plasma metabolome, and epigenetic modifications in subjects diagnosed with metabolic syndrome. Participants were administered either allogeneic FMT derived from lean donors or autologous FMT, with outcomes evaluated prior to and six weeks subsequent to the transplantation procedure. The administration of allogeneic FMT precipitated a more pronounced alteration in gut microbiota, facilitating the introduction of novel species that modulated the plasma metabolome and reconfigured DNA methylation patterns in peripheral blood mononuclear cells. Importantly, the presence of Prevotella amplicon sequence variants exhibited a correlation with the methylation status of the AFAP1 gene, which is instrumental in the regulation of mitochondrial functionality and insulin sensitivity, and was linked to enhanced peripheral glucose uptake. Collectively, FMT not only transformed the gut microbiota but also exerted an influence on host metabolic processes and the epigenetics of immune cells, underscoring its prospective utility in augmenting insulin sensitivity and providing novel perspectives for interventions targeting metabolic syndrome (van der Vossen et al., 2021).

### 5.2 Molecular and cellular mechanisms

#### 5.2.1 mtDNA as a central mediator linking microbiota and immunity

Accumulating evidence suggests that mitochondrial dysfunction serves as a critical intermediary between gut microbiota-derived signals and host immune responses. Dysbiosis and microbial metabolites, including TMA, TMAO, PAGln, and LPS, can induce oxidative stress, mitochondrial injury, and perturbations in mtDNA homeostasis (Bordoni et al., 2024; Yang et al., 2025; Zhang et al., 2025; Zhao et al., 2025). Although not all studies directly measured mtDNA release, alterations in mtDNA copy number, methylation status, and mitochondrial integrity indicate conditions that may favor mtDNA leakage into the cytosol. Due to its bacterial ancestry and the presence of unmethylated CpG motifs, mtDNA acts as a potent DAMP capable of initiating innate immune signaling pathways (West et al., 2015).

#### 5.2.2 mtDNA-mediated activation of cGAS–STING signaling

Cytosolic mtDNA is recognized by cGAS, which catalyzes the formation of cyclic GMP–AMP and subsequently activates STING signaling (West et al., 2015). Activation of the cGAS–STING pathway promotes downstream TBK1–IRF3 and NF-κB signaling, resulting in type I interferon production and inflammatory cytokine release. Evidence from the microbiota–gut–mammary axis study demonstrated that elevated mtDNA levels activated cGAS–STING signaling and contributed to inflammatory injury in mammary tissues (Zhao et al., 2025). These findings support the concept that mtDNA serves as a mechanistic bridge between microbiota-induced mitochondrial stress and systemic immune activation.

#### 5.2.3 mtDNA-dependent NLRP3 inflammasome activation

In parallel with cGAS–STING signaling, oxidized mtDNA functions as a potent activator of the NLRP3 inflammasome (Shimada et al., 2012). Microbiota-associated metabolites and inflammatory stimuli promote mtROS generation and mitochondrial membrane damage, thereby increasing the likelihood of mtDNA release (Zhang et al., 2025). Once present in the cytosol, mtDNA facilitates NLRP3 inflammasome assembly, caspase-1 activation, and maturation of IL-1β and IL-18. Consequently, mtDNA acts as a co-activator of both cGAS–STING and NLRP3 pathways, amplifying inflammatory responses and linking mitochondrial dysfunction to microbiota-driven immune dysregulation.

#### 5.2.4 Therapeutic implications of targeting the microbiota–mtDNA axis

Several interventions discussed in this section, including probiotics, prebiotics, postbiotics, butyrate supplementation, and FMT, demonstrated the capacity to restore microbial balance, improve mitochondrial function, reduce oxidative stress, and preserve mtDNA integrity (van der Vossen et al., 2021; Chen et al., 2022; Dunislawska et al., 2023; Zhang et al., 2025; Murgina et al., 2026). By limiting mitochondrial damage and preventing excessive mtDNA-mediated signaling, these approaches may attenuate activation of both cGAS–STING and NLRP3 pathways. Collectively, these findings highlight the therapeutic potential of targeting the microbiota–mitochondria–mtDNA axis for the management of inflammatory, metabolic, neurodegenerative, and infectious diseases.

## 6 Therapeutic modulation of the microbiome–mitochondria axis

The targeting of the microbiome–mitochondria axis has emerged as a highly promising approach for the modulation of host immune responses and inflammatory processes in the context of infectious diseases. Interventions designed to restore microbial equilibrium or enhance mitochondrial functionality have the potential to mitigate excessive inflammation while simultaneously facilitating the clearance of pathogens (Kho and Lal, 2018). The reciprocal communication between microbial metabolites and mitochondrial signaling pathways offers numerous therapeutic avenues for exploration (Koh et al., 2016). These interventions exert significant influence on mitochondrial bioenergetics, ROS production, and the activation of inflammasomes, thereby determining immune outcomes (Marchi et al., 2014). Notably, the association between dysbiosis and mitochondrial dysfunction has been implicated in chronic infections, sepsis, and immune dysregulation, underscoring the necessity for integrative therapeutic strategies. Recent advancements in systems biology and multi-omics methodologies are expediting the identification of novel targets within this axis, thereby paving the way for more effective and personalized therapeutic interventions aimed at restoring immune homeostasis.

### 6.1 Probiotics, postbiotics, and phytochemicals

Probiotics, postbiotics, and phytochemicals are pivotal modulators of the microbiome–mitochondria axis due to their capacity to affect microbial composition and mitochondrial functionality. Probiotics are defined as live microorganisms that provide health advantages to the host when administered in sufficient quantities, whereas postbiotics are characterized as non-viable microbial products or metabolites that exert biological effects independently of the administration of living organisms. Phytochemicals encompass bioactive compounds derived from botanical sources, including polyphenols, flavonoids, and alkaloids, which modulate host metabolism, immune responses, and the ecological dynamics of microbial communities. Despite their distinct origins, these three categories exhibit interconnections through their ability to influence gut microbial diversity, the production of microbial metabolites, mitochondrial functionality, and the pathways involved in inflammatory signaling (Hill et al., 2014; Salminen et al., 2021). Probiotics such as Lactobacillus and Bifidobacterium enhance the integrity of the epithelial barrier and mitigate inflammation, in part by modulating mitochondrial ROS and energy metabolism (Marco et al., 2021). Postbiotics, such as SCFAs including butyrate, directly influence mitochondrial respiration and suppress excessive activation of the NLRP3 inflammasome (Louis et al., 2014). Phytochemicals like polyphenols (for instance, resveratrol and curcumin) further facilitate mitochondrial biogenesis and bolster antioxidant defenses through the activation of signaling pathways such as AMPK and SIRT1 (Baur and Sinclair, 2006). Collectively, these interventions enhance host defense mechanisms while minimizing tissue damage during infectious processes. Their multi-faceted mechanisms render them appealing candidates for adjunctive therapies, particularly in pathological conditions characterized by dysbiosis and mitochondrial dysfunction.

### 6.2 Mitochondrial-targeted therapies

Mitochondrial-targeted therapeutic approaches are designed to restore the structural and functional integrity of mitochondria, mitigate oxidative stress, and modulate the signaling pathways associated with immune responses. Pharmacological agents, such as mitochondria-targeted antioxidants (for instance, MitoQ and SkQ1), exhibit selective accumulation within mitochondria, thereby neutralizing excessive ROS and preventing oxidative cellular damage (Smith et al., 2011). Empirical evidence has demonstrated that these compounds can attenuate the activation of the NLRP3 inflammasome and diminish the production of pro-inflammatory cytokines in preclinical experimental models (Dashdorj et al., 2013). Pharmacological agents such as mitochondria-targeted antioxidants (e.g., MitoQ and SkQ1) selectively accumulate within mitochondria and neutralize excessive mtROS. For example, MitoQ significantly ameliorated DSS-induced colitis and suppressed NLRP3 inflammasome activation through reduction of mitochondrial oxidative stress. Furthermore, MitoQ protected against severe burn-induced liver injury by preserving mitochondrial integrity and inhibiting the mtDNA–NLRP3 signaling axis. These findings support the therapeutic potential of mitochondria-targeted antioxidants in controlling inflammasome-driven inflammatory responses (Dashdorj et al., 2013; Wu et al., 2020). Furthermore, modulators of mitochondrial dynamics and mitophagy, which include AMPK activators and PINK1/parkin enhancers, facilitate the elimination of damaged mitochondria and contribute to the maintenance of cellular homeostasis (Herzig and Shaw, 2018). Such interventions not only enhance mitochondrial functionality but also regulate innate immune responses, positioning them as promising therapeutic strategies for the management of infectious and inflammatory disorders. The ongoing development of mitochondria-specific pharmacological agents presents novel opportunities to target the mechanistic underpinnings of disease at the organelle level.

### 6.3 Implications for precision medicine

The incorporation of microbiome and mitochondrial profiling within precision medicine paradigms presents novel avenues for the development of customized therapeutic approaches. The heterogeneity in microbiota composition and mitochondrial functionality significantly affects individuals’ vulnerability to infections and their responses to treatments (Shukla et al., 2024). Tailored interventions can enhance the efficacy of probiotics, dietary components, and mitochondrial-focused therapies, contingent upon the unique biological signatures of each patient (Hasin et al., 2017). Specific biomarkers identified in the studies summarized in **Tables 1**, **2** may support precision-medicine approaches. For example, elevated circulating mtDNA levels may identify patients with enhanced mitochondrial stress and inflammasome activation (Zhao et al., 2025), whereas increased TMAO concentrations may indicate individuals at greater risk of inflammatory and cardiometabolic complications (Wu et al., 2020; Chang et al., 2025). Likewise, alterations in SCFA profiles, particularly reductions in acetate and butyrate-associated signaling, may help identify patients who are more likely to benefit from microbiota-targeted interventions such as probiotics, postbiotics, or fecal microbiota transplantation (Wang et al., 2025; Yu et al., 2025). The integration of these biomarkers with microbiome, mitochondrial, and immune profiling may improve patient stratification, therapeutic selection, and treatment monitoring. More specifically, the biomarkers elucidated within this review, encompassing circulating mtDNA levels, TMAO concentrations, and SCFA profiles, may yield clinically pertinent information pertinent to patient stratification. Elevated mtDNA levels may signify ongoing mitochondrial damage and inflammasome activation, thus identifying individuals who could potentially benefit from mitochondria-targeted therapies or NLRP3 inhibition. Similarly, augmented TMAO concentrations may pinpoint patients at an elevated risk for cardiometabolic and inflammatory complications, whereas diminished SCFA levels may indicate disrupted microbial homeostasis and the prospective necessity for microbiota-directed interventions such as probiotics, postbiotics, dietary fiber supplementation, or FMT. The amalgamation of these biomarkers with microbiome and mitochondrial profiling could enhance the precision of individualized therapeutic decision-making. Moreover, the precise targeting of the microbiome–mitochondria interface has the potential to mitigate adverse effects and enhance clinical outcomes by adapting treatments to the fundamental pathophysiological processes. This methodology signifies a transformative shift towards a more predictive, preventive, and individualized approach in the management of infectious and inflammatory disorders.

## 7 Clinical relevance and translational perspectives

Clinical investigations, although still emerging, have already provided encouraging translational evidence. Preliminary studies employing FMT in metabolic syndrome have demonstrated improvements in insulin sensitivity, microbial diversity, and host metabolic profiles, while experimental and early clinical observations in sepsis suggest that microbiota restoration may improve intestinal barrier integrity, reduce systemic inflammation, and enhance clinical outcomes. These findings support the microbiome–mitochondria axis as a promising therapeutic target for future clinical development. Additionally, circulating mtDNA concentrations and inflammasome-associated markers have been posited as promising biomarkers indicative of disease severity and therapeutic efficacy, thereby presenting opportunities for tailored interventions. Nevertheless, numerous obstacles persist concerning effective translation. Variability among individuals in microbiome composition, strain-specific probiotic effects, and contextually dependent mitochondrial responses hinder the ability to generalize findings. Furthermore, there exists a paucity of mechanistic investigations that establish connections between specific microbial taxa and the release of mtDNA as well as the activation of inflammasomes in human subjects. To address these limitations, future studies should incorporate multicenter stratified randomized controlled trials that account for baseline microbiota composition, host metabolic status, and inflammatory phenotypes. In addition, microbiota-type-based adaptive trial designs may facilitate the identification of patient subgroups most likely to benefit from targeted microbiome or mitochondrial interventions. The integration of multi-omics datasets, including microbiome, metabolome, mitochondrial, and immune profiling, will further improve mechanistic interpretation and patient stratification. The standardization of interventions, the implementation of comprehensive multi-omics analyses, and the execution of longitudinal clinical trials are crucial for elucidating causal relationships and enhancing therapeutic approaches. Subsequent research should concentrate on the integration of microbiome, mitochondrial, and immune biomarkers to formulate precision-targeted interventions, thereby utilizing the microbiome–mitochondria axis to effectively alleviate infectious and inflammatory diseases.

## 8 Conclusions

The emerging evidence reviewed here highlights the microbiome–mitochondria axis as a critical regulator of host immunity and inflammatory responses. Among the proposed mechanisms, mtDNA appears to function as a central signaling hub linking microbiota dysbiosis, mitochondrial dysfunction, and inflammasome activation, while microbiota-derived metabolites, including SCFAs, TMAO, and indole derivatives, exert bidirectional effects on mitochondrial homeostasis and immune regulation. Furthermore, therapeutic approaches targeting PINK1/Parkin-mediated mitophagy, mitochondrial quality control, and NLRP3 inflammasome signaling have shown considerable promise in preclinical models of infectious and inflammatory diseases. However, it is important to recognize that many currently reported associations between dysbiosis, mitochondrial dysfunction, and inflammasome activation remain incompletely validated as direct causal relationships. In addition, the translational interpretation of existing evidence is limited by the heterogeneity of experimental models, differences in microbiome profiling methodologies, and the limited availability of validated clinical biomarkers capable of reliably reflecting microbiome–mitochondria interactions. Addressing these challenges through mechanistic studies and clinically relevant validation frameworks will be essential for translating this rapidly evolving field into effective therapeutic interventions.
